# Methylenetetrahydrofolate (MTHFR), the One-Carbon Cycle, and Cardiovascular Risks

**DOI:** 10.3390/nu13124562

**Published:** 2021-12-20

**Authors:** Shanel Raghubeer, Tandi E. Matsha

**Affiliations:** SAMRC/CPUT/Cardiometabolic Health Research Unit, Department of Biomedical Science, Faculty of Health & Wellness Sciences, Cape Peninsula University of Technology, Cape Town 7530, South Africa; matshat@cput.ac.za

**Keywords:** MTHFR, gene polymorphisms, C677T, A1298C, vitamin B, cardiovascular diseases, retinopathy, inflammation, folate

## Abstract

The 5-10-methylenetetrahydrofolate reductase (MTHFR) enzyme is vital for cellular homeostasis due to its key functions in the one-carbon cycle, which include methionine and folate metabolism and protein, DNA, and RNA synthesis. The enzyme is responsible for maintaining methionine and homocysteine (Hcy) balance to prevent cellular dysfunction. Polymorphisms in the *MTHFR* gene, especially C677T, have been associated with various diseases, including cardiovascular diseases (CVDs), cancer, inflammatory conditions, diabetes, and vascular disorders. The C677T *MTHFR* polymorphism is thought to be the most common cause of elevated Hcy levels, which is considered an independent risk factor for CVD. This polymorphism results in an amino acid change from alanine to valine, which prevents optimal functioning of the enzyme at temperatures above 37 °C. Many studies have been conducted to determine whether there is an association between the C677T polymorphism and increased risk for CVD. There is much evidence in favour of this association, while several studies have concluded that the polymorphism cannot be used to predict CVD development or progression. This review discusses current research regarding the C677T polymorphism and its relationship with CVD, inflammation, diabetes, and epigenetic regulation and compares the evidence provided for and against the association with CVD.

## 1. Introduction

5-10-Methylenetetrahydrofolate reductase (MTHFR) is an essential enzyme in folate and homocysteine (Hcy) metabolism. The gene for this enzyme is located on the short arm of chromosome 1 (1p36.3) and encodes for dimeric proteins [1]. The major product of the *MTHFR* gene is a 77 kDa protein, with a second 70 kDa isoform found in humans [2]. MTHFR catalyses the irreversible reduction of 5-10-MTHF to 5-methylTHF, a circulatory form of folate used in the remethylation of Hcy to methionine [2,3]. In total, 34 rare but deleterious mutations have been reported, and the location of the mutation may influence its impact [4]. For example, relative to their size, exons 5 and 6 harbour the most mutations, which may be more deleterious since these exons are located within the catalytic domain and may play a role in substrate binding [4]. Mudd et al. published the first report on MTHFR involvement in disease upon identifying a patient with homocystinuria due to severe MTHFR deficiency [5]. In 1988, a thermolabile variant of the MTHFR enzyme was identified in patients with cardiovascular disease (CVD). This deficiency was milder but more common and induced mild elevation of Hcy levels, which may be an independent risk factor for CVD [6].

The most common cause of elevated Hcy levels is thought to be the C677T *MTHFR* polymorphism (rs 1801133). This polymorphism involves the substitution of cytosine with thymine at position 677, resulting in an amino acid change from alanine to valine in the enzyme [1,7,8]. This common *MTHFR* gene mutation affects Hcy levels and is thought to contribute to hyperhomocysteinemia, reduced folate levels, and several CVD-associated diseases [9]. The C677T mutation reportedly disrupts thermostability, leading to enzyme dysfunction [10]. This product is termed thermolabile since enzyme activity is reduced at temperatures above 37 °C [11]. MTHFR enzyme activity appeared 50–60% lower in C677T homozygous (TT) patients, with activity decreasing by 65% at 46 °C [10]. Another commonly encountered polymorphism occurs at position 1298 on the *MTHFR* gene and involves the substitution of adenine with cytosine, resulting in an amino acid change from glutamate to alanine in the enzyme [12]. This mutation also produces reduced enzyme activity although not to the same extent as that caused by the C677T polymorphism [12].

Researchers investigating the ethnic and geographical distribution of the C677T *MTHFR* gene polymorphism in new-borns across 16 regions of Europe, Asia, Australia, the Middle East, and the Americas reported that the TT genotype was more frequently observed in China, Mexico, and Southern Italy, with a particularly low frequency observed in new-borns of African ancestry [13]. Furthermore, Schneider et al. reported that the frequency of the C677T polymorphism was lowest in Africa compared to that in populations from Europe and Asia [14]. Therefore, the prevalence of the C677T polymorphism is dependent on ethnicity and geographical location, with increased prevalence reported in Italian and Hispanic populations and low prevalence reported in African-American and Sub-Saharan African populations [12]. The MTHFR enzyme is involved in the transmethylation pathway, which involves the conversion of Hcy to methionine [15]. Research has suggested a connection between *MTHFR* gene polymorphisms, especially C677T and A1298C (rs 1801131), and several disease states, including kidney disease, CVD, neural tube defects, cancer, and liver disease [16]. This review serves to discuss the current research conducted in this field and to observe the evidence for and against the association between *MTHFR* gene polymorphisms and CVD.

## 2. MTHFR in Inflammation

Inflammation is mediated primarily by the nuclear factor kappa-light-chain-enhancer of activated B-cells (NF-κB) pathway, which involves several related transcription factors. The NF-κB protein is sequestered and rendered inactive in the cytoplasm during normal conditions by the inhibitor of nuclear factor kappa B alpha (IκBα) protein [17]. NF-κB is activated by certain signals, such as reactive oxygen species (ROS) and pro-inflammatory cytokines (interleukin (IL)-1, IL-6, and tumour necrosis factor alpha (TNFα)), and translocates into the nucleus to activate the inflammatory cascade, thus regulating the inflammatory response [17,18]. Inflammation occurs to remove potentially harmful stimuli, such as pathogens, toxins, or damaged cells, and promote tissue homeostasis [19]. However, if acute inflammation is not resolved, chronic inflammation, which is associated with the development and progression of numerous illnesses, may occur [19]. C-reactive protein (CRP) is an acute-phase protein primarily synthesised by hepatocytes and to a lesser extent by smooth muscle cells, lymphocytes, and adipocytes [20]. Increasing evidence indicates that CRP plays a role in inflammatory responses and other immune system responses, such as apoptosis and the production of cytokines, especially TNFα and IL-6. Further, CRP has been shown to increase up to 1000-fold at infection or inflammation sites and has been associated with CVDs [20]. Several factors can influence CRP levels, including weight, lipid profile, age, gender, and smoking status, as well as polymorphisms in the *CRP* gene [21]. Diabetes patients with the C677T polymorphism have reportedly exhibited increased levels of CRP and IL-6 [22].

Regarding the *MTHFR* C677T polymorphism and inflammation, Dedoussis et al. reported an association between the homozygous mutant genotype (TT) and increased inflammatory markers [23]. The study investigated inflammatory markers and *MTHFR* C677T genotype association in 574 CVD-free subjects from the Attica region in Greece and reported that 11% of subjects presented with the homozygous TT genotype, 48% with the heterozygous CT genotype, and 41% with the homozygous CC genotype (normal). Inflammatory markers, including CRP, white blood cell counts, and amyloid-A levels, appeared elevated in the TT genotype subjects compared to the CC and CT genotype individuals despite controlling for confounding variables [23]. Mild MTHFR deficiency caused by the C677T polymorphism was found to affect inflammatory and lipid pathways in a murine model [24]. Researchers found that MTHFR deficiency reduced s-adenosylmethionine (SAM) and betaine levels and increased s-adenosylhomocysteine (SAH) levels, resulting in reduced methylation capacity and alteration of inflammatory mediators. Furthermore, MTHFR-deficient mice fed a high-fat diet exhibited exacerbated effects in inflammatory and lipid pathways, with increased inflammation and lipid accumulation. These results suggest that MTHFR deficiency coupled with a low-quality high-fat diet may increase the risk of developing non-alcoholic fatty liver disease (NAFLD) due to disrupted methylation capacity, lipid metabolism, and inflammatory responses [24]. An estimated one billion people have NAFLD [25], which is characterised by inflammation and ectopic fat in the liver, causing liver dysfunction, fibrosis, end-stage liver disease, and cirrhosis [26]. Research has provided evidence highlighting the influence of microRNAs (miRNA) on NAFLD [27,28,29,30]. MicroRNAs are short, non-coding RNA molecules that function to regulate the expression of messenger RNA (mRNA), thereby influencing gene expression and protein synthesis [26]. An et al. investigated the effect of the C677T *MTHFR* polymorphism and miR-149 on NAFLD. Results showed that miR-149 was upregulated in TT genotyped hepatocytes treated with long-chain fatty acid (FFA), while *MTHFR* was downregulated in these hepatocytes [26]. However, FFA did not affect hepatocytes genotyped as CC, suggesting that the polymorphism influences the response to FFA and the development of NAFLD. miR-149, a regulator of the *MTHFR* gene, was upregulated in response to FFA in TT genotyped hepatocytes and in turn decreased *MTHFR* expression [26]. This study suggests that *MTHFR* genotype affects susceptibility to NAFLD.

The *MTHFR* C677T polymorphism was shown to be associated with inflammatory diseases, such as psoriasis (Caucasian Czech population) [31] and inflammatory bowel disease (Caucasian Irish population) [32], while both C677T and A1298C variants have been linked to rheumatoid arthritis in a Caucasian and Asian population [33,34]. Systemic inflammation has been linked with several diseases, including diabetes, coronary artery disease, and cancer. Studies have shown the presence of inflammatory processes in pancreatic beta cells of diabetes patients [35], and insulin resistance has been linked to cytokine-induced inflammation [36]. Furthermore, inflammatory cells and cytokines (TNFα, IL-1, IL-6, and monocyte chemoattractant protein-1 (MCP-1)) are thought to play important roles in the development and progression of atherosclerosis [37]. Khalighi et al. investigated whether *MTHFR* gene polymorphisms contributed to systemic inflammation in a cohort of 292 patients by determining two markers of systemic inflammation, namely neutrophil-to-lymphocyte ratio (NLR) and platelet-to-lymphocyte ratio (PLR) [37]. This study reported that the A1298C variant produced opposite effects compared to the C677T variant. Results showed that patients with the C677T variant displayed a greater NLR than C677T wild-type patients, while those with the A1298C variant displayed lower NLR and PLR than A1298C wild-type patients [37]. The above-mentioned studies provide compelling evidence that suggests a link between systemic inflammation and *MTHFR* gene polymorphisms.

## 3. Vitamin B Pathway and MTHFR Effects

**Figure 1:**
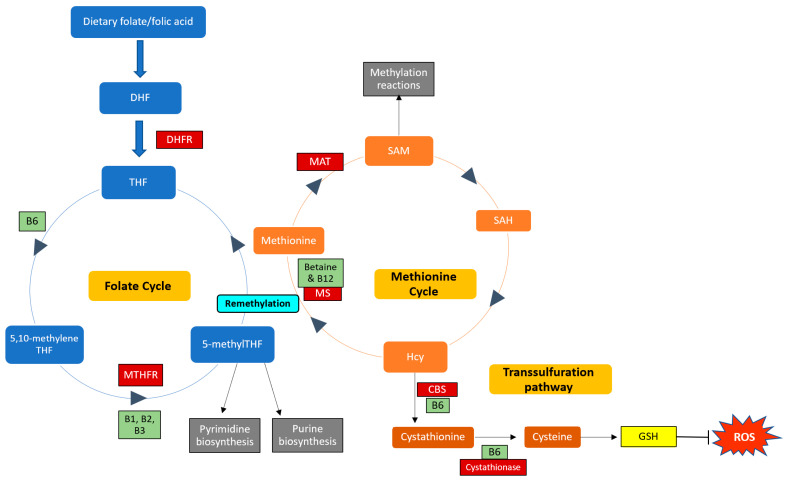
Enzymes and cofactors involved in the one-carbon cycle, including methionine and folate metabolism. Dietary folate (vitamin B9) is converted into dihydrofolate (DHF) by the enzyme dihydrofolate reductase (DHFR) and is then reduced to tetrahydrofolate (THF). THF is converted to 5,10-methyleneTHF, which is converted to 5-methylTHF by the MTHFR enzyme (using vitamins B1, B2, and B3 as cofactors). 5-methylTHF is then used as a methyl donor in pyrimidine and purine synthesis and can donate a methyl group to regenerate methionine from homocysteine (Hcy); this reaction is catalysed by methionine synthase (MS) with vitamin B12 as a cofactor. This is termed remethylation. Dietary betaine from the liver can also serve as a methyl donor and participate in remethylation. Thereafter, methionine adenosyltransferase (MAT) catalyses the transfer of adenosine to methionine to generate s-adenosylmethionine (SAM), which functions in methylation reactions. SAM is then demethylated and forms s-adenosylhomocysteine (SAH), which is hydrolysed to form Hcy. Hcy can now enter the transsulfuration pathway to form cystathionine (catalysed by cystathionine beta-synthase (CBS)) and cysteine (catalysed by cystathionase). Cysteine is then used to synthesise glutathione (GSH), thereby regenerating antioxidant levels to combat damage by reactive oxygen species (ROS). Enzymes and cofactors are indicated in dark red and light green squares, respectively.

B vitamins play important roles in the one-carbon cycle (Figure 1), which is essential for cellular function. The one-carbon cycle consists of interlinking biochemical pathways that include folate and methionine metabolism [38]. These metabolic pathways generate methyl groups for several essential processes, including DNA synthesis, antioxidant generation, and amino acid homeostasis [39]. Furthermore, methyl groups are involved in methylation reactions in epigenetic regulation, thereby influencing gene expression and protein synthesis [40]. One-carbon metabolism is regulated by methionine, vitamin B12, and vitamin B9 (folate), which are essential dietary requirements [39]. Folate and methionine cycles are linked by a rate-limiting enzyme, methionine synthase (MS), which converts Hcy to methionine using 5-methylTHF (produced by MTHFR) and B12 [41]. Ultimately, this cycle produces methyl donors that participate in essential cellular processes [42]. Therefore, genetic polymorphisms and vitamin B deficiencies can be detrimental to the one-carbon cycle, which may result in Hcy accumulation and vascular endothelium damage [43].

The C677T and A1298C polymorphisms are thought to be primary contributors to hyperhomocysteinemia, resulting in an increased risk of developing thrombosis [44]. Xuan et al. reported that the C677T polymorphism is associated with a 50% reduction in MTHFR enzyme activity as well as increased plasma Hcy and reduced plasma folic acid concentrations, which may promote endothelial dysfunction and increase the risk of developing various CVDs [45]. Characteristics of MTHFR enzyme dysfunction include elevated plasma Hcy levels, which is often used as the first indicator, increased plasma SAH and cystathionine, and decreased methionine and SAM [3]. These imbalances are attributed to dysfunctional methionine metabolism, which may further influence DNA methylation and gene expression. MTHFR-deficient patients may present with developmental delays, neurological and vascular issues, seizures, or thrombosis [2]. Severe MTHFR deficiencies are rare but are also the most commonly encountered inborn errors of folate metabolism [2].

Research has reported an association between hyperhomocysteinemia and low bone mineral density (BMD) as well as CRP, vitamin D, vitamin B12, and folate levels in postmenopausal women [46]. Two hundred and fifty women (aged 50–65 years) were recruited and divided into two groups based on BMD. Of the 250 women, 155 had low BMD, and 97 women were within the normal BMD range. Women with low BMD presented with increased Hcy and CRP levels and decreased levels of vitamin B12, vitamin D, and folate [46]. Furthermore, 77% of women with low BMD presented with the C677T *MTHFR* polymorphism, while only 37% of women with normal BMD presented with the mutation. Taken together, these results suggest an association between the C677T *MTHFR* polymorphism and elevated Hcy levels and inflammation, which may further influence osteoporosis onset [46]. The MTHFR enzyme is involved in the formation of SAM, which donates a methyl group for the progression of reactions catalysed by catechol-O-methyltransferase (COMT), an enzyme that participates in hormone regulation, thereby inactivating catechol oestrogens and playing a role in oestrogen metabolism [47]. Catechol oestrogens may contribute to increased ROS production, thereby inducing DNA damage, oxidative cell damage, and inflammation [48], and may further influence carcinogenesis and the development of CVDs and related diseases [49,50]. Research has shown that oestrogen-induced hypertension is age- and sex-specific; therefore, polymorphisms in the *MTHFR* gene may contribute to disrupted oestrogen metabolism and in turn influence oestrogen-associated diseases [49].

Vitamin B12 deficiency is thought to contribute to increased Hcy levels. Patients with low folic acid levels often present with low levels of B12 and other B vitamins [51]. In a study investigating the relationship between folate and homocysteine levels, sub-normal folate levels (<2 ng/mL) resulted in two-fold increases in Hcy serum levels, while low folate levels (2–3.9 ng/mL) resulted in increased Hcy levels compared to patients with normal folate levels (5–17.9 ng/mL) [51]. Therefore, folic acid and vitamin B12 supplementation is thought to improve hyperhomocysteinemia. Several studies have reported higher Hcy levels in patients with the TT genotype compared to CC, often coupled with low plasma folic acid levels [52,53,54]. Pereira et al. reported that plasma Hcy levels appeared increased in TT vs. CC patients (16.2 vs. 8.2 µmol/L), while low vitamin B12 levels were observed in TT vs. CC patients (196 vs. 301 pmol/L) [55]. Moreover, folate deficiency results in thymidine depletion, which increases the incorporation of uracil into DNA, resulting in impaired DNA repair and an increased likelihood of malignant transformation due to DNA instability and chromosome aberrations [56].

## 4. Effects Related to Cardiovascular Diseases

Many studies have reported associations between *MTHFR* gene polymorphisms, increased Hcy levels, and cardiovascular complications. A meta-analysis by Kang et al. reported an association between increased plasma Hcy and increased risk for haemorrhagic stroke [57]. Klerk et al. reported that patients with the C677T polymorphism presented with an increased risk of developing heart disease [58], and Yang et al. observed an increased risk for diabetic nephropathy development in a Caucasian population with type 2 diabetes mellitus (T2DM) and C677T polymorphisms [59], while Frosst et al. suggested that the C677T mutation may play a role in vascular diseases [7]. Kluijtmans et al. reported that the homozygous C677T *MTHFR* gene polymorphism is associated with a three-fold increased risk for the premature development of CVD [60]. However, conflicting evidence also exists. Zhong et al. reported no association with diabetes development in Asian, Caucasian, and African cohorts [61].

Bahadir et al. assessed the implications of the *MTHFR* C677T gene polymorphism in CVD development in diabetes patients. Patients with T2DM and severe CVD (107) but without nephropathy or retinopathy were recruited for the study [15]. The researchers employed polymerase chain reaction-restriction fragment length polymorphism (PCR-RFLP) to determine the presence of *MTHFR* C677T polymorphisms and reported 31, 62, and 14 subjects with the CC, CT, and TT genotypes, respectively. The results reported no correlation between *MTHFR* genotypes and several measured parameters, including visceral fat area, diabetes duration, cholesterol and triglyceride profiles, vitamin B12, Hcy, and HbA1c levels, and fasting plasma glucose [15]. This study concluded that the *MTHFR* C677T polymorphism cannot be used to assess CVD risk in diabetes patients [15]. However, the small sample size should be noted along with the lack of lifestyle information, such as smoking status, alcohol consumption, and information pertaining to dietary habits and physical activity.

Incidence of thrombosis further aggravates CVDs and associated disorders. Hyperhomocysteinemia has been associated with increased incidence of venous thrombosis. However, research has provided conflicting reports as to whether the C677T *MTHFR* polymorphism contributes to thrombosis [62]. Some researchers have reported no association between the C677T polymorphism and thrombosis [62,63], while others have reported significant associations between the two, or reported that the polymorphism is a risk factor for thrombosis development [64,65,66,67].

Several studies have suggested that *MTHFR* gene variants may contribute to disruptions in the Hcy pathway, which in turn exacerbates T2DM-associated complications [68,69]. High homocysteine levels were reportedly associated with increased risk of CVD in diabetes patients by promoting endothelial dysfunction and increasing the likelihood of atherosclerosis development [70]. Xuan et al. reported an association between the C677T polymorphism and myocardial infarction (MI) risk, but Alizadeh et al. reported no overall association in the TT vs. CT model [44,45]. MI is one of the most prevalent CVDs, and occurs when coronary arteries are occluded by clots produced during atherosclerotic plaque rupture [71]. MI is complex, often involves genetic and environmental factors, and is thought to be influenced by Hcy levels [44]. Therefore, the association between MTHFR polymorphisms and MI has been a research focus in recent years. The meta-analysis conducted by Alizadeh et al. included 47 studies and found no statistically significant association between MTHFR polymorphisms and MI risk [44]. However, sub-group analysis by ethnicity revealed that the T allele increased MI risk by 63% in African populations vs. the C allele, whereas the CT genotype decreased MI risk in North American (Caucasian) populations (vs. the CC genotype) [44]. Furthermore, the C677T polymorphism conferred protection against MI in the elderly (>50 years of age). The discrepancy between the two studies may be due to the sample size and controls used or may be because the meta-analysis by Xuan et al. was conducted in 2011, while that by Alizadeh et al. was conducted in 2016, and during this time, many more studies were conducted regarding the C677T *MTHFR* gene polymorphism and MI risk, ultimately producing conflicting results.

The C677T polymorphism is thought to be an independent risk factor for hypertension. Nishio et al. conducted a study in 1996 investigating the frequency of the C677T *MTHFR* polymorphism in relation to hypertension in a Japanese population and found no significant relationship between the two [72]. However, Qian et al. performed a meta-analysis of 25 studies investigating the polymorphism in more than 2800 Caucasian and Asian individuals with hypertension and reported a significant association between the polymorphism and hypertension in both populations [73]. Furthermore, a meta-analysis conducted by Meng et al. reported that the C677T *MTHFR* polymorphism is associated with T2DM susceptibility in Asian population but not in African or Caucasian populations [74]. Further reading regarding meta-analysis studies can be found in Table 1.

## 5. DNA Methylation and *MTHFR* Polymorphisms

DNA methylation, which is vital for normal gene regulation, involves the addition of a methyl group to cytosine nucleotides and is catalysed by DNA methyltransferases [102]. DNA methylation functions to regulate gene transcription by preventing transcription factor binding or promoting the binding of methyl-binding proteins, thus inhibiting or reducing gene expression [103]. Genetic polymorphisms and vitamin B deficiencies negatively impact the one-carbon cycle, which converts Hcy to methionine for DNA methylation and the maintenance of healthy vasculature [43]. The methyl group necessary for methylation can be donated by the Hcy cycle via methionine metabolism. Therefore, MTHFR is important in the process of methylation, as it participates in the conversion of methionine into SAM, a universal methyl radical donor [104].

Type 2 diabetes mellitus (T2DM) develops as a result of many interacting factors, including environmental and genetic aspects. Several studies have shown that DNA methylation patterns appear altered in diabetes patients [105,106,107], and this has prompted extensive research into epigenetic modifications in diabetes patients as well as the influence of genetic polymorphisms, such as the C677T and A1298C *MTHFR* gene polymorphisms [108]. Matsha et al. investigated global DNA methylation in a mixed-ancestry population from South Africa and showed that global DNA methylation increased in prediabetic and diabetic subjects, with a more pronounced increase in screen-detected diabetes subjects [108]. Furthermore, this research revealed that DNA methylation was not associated with the *MTHFR* C677T polymorphism but was instead associated with the *NOS3* G894T polymorphism in this population. The *NOS3* gene has been linked with diabetes and nitric oxide (NO) bioavailability, which influences endothelial function and contributes to vascular complications in diabetes [108]. This research provided important information regarding the influence of genetic and epigenetic changes on non-communicable diseases in the African setting since studies pertaining to this topic are lacking in Africa.

Several studies have suggested that hyperhomocysteinemia contributes to the development of diabetic nephropathy (DN) and retinopathy (DR) in patients with diabetes, especially those with *MTHFR* polymorphisms [109,110]. Diabetic retinopathy is a commonly encountered microvascular complication in diabetes patients and is the leading cause of adult-onset blindness worldwide [111]. Nitric oxide and ROS are known to play pivotal roles in the onset and progression of DR [43]. One-carbon cycle disruption, vitamin B deficiencies, and Hcy accumulation reportedly contribute to the development and progression of DR [43]. Diabetic nephropathy is a diabetes-associated complication and often leads to end-stage renal disease (ESRD), culminating in kidney failure [112]. The pathogenesis of DN is complex and involves an interplay between environmental and genetic factors, such as familial aggregation and the presence of polymorphisms [113]. The MTHFR gene is thought to play an important role in DN, since the MTHFR enzyme is involved in the metabolism of folate, which is lost to a greater extent during dialysis [114]. The C677T and A1298C polymorphisms are reportedly associated with DN and ESRD progression in a South Indian population. However, homocysteine levels did not correlate with ESRD progression in DN in this population [113]. In contrast with other studies, Errera et al. found no association between the C677T *MTHFR* gene polymorphism and the severity or progression of DR in a Brazilian population while excluding the influence of African ancestry from the data analysis. The authors suggest that the C677T *MTHFR* polymorphism is not a useful predictive marker for diabetes or DR [115].

Dos Santos Nunes et al. recruited 105 individuals to study the influence of methylation of the *MTHFR* gene promoter region on diabetes complications, such as DR and DN [116]. In total, 16 diabetes patients with DR and 29 with DN were recruited, while 60 diabetes patients without complications served as control subjects. Results of this study showed that hypermethylation in the *MTHFR* gene promoter region was associated with DR, in addition to biochemical, oxidative stress, and inflammatory parameters related to T2DM and its associated complications [116]. The CpG sites investigated in this study were located near transcription factor binding sites, and hypermethylation of these sites may disrupt MTHFR enzyme functions, thereby affecting Hcy and methionine metabolism [116]. This epigenetic dysregulation may exacerbate enzyme dysfunction and promote endothelial cell damage, further impacting microvasculature complications of diabetes, such as DR and DN. However, further research should be conducted on this topic using larger cohorts.

## 6. Treatments and Therapeutic Options

Vitamin B supplementation is particularly important during pregnancy if either parent is homozygous for the C677T *MTHFR* gene polymorphism, as this polymorphism has been linked with increased incidence of neural tube defects related to low folate status [117,118]. Betaine is often used as a treatment for MTHFR deficiency. Research has shown that betaine supplementation protects against neurocognitive decline in MTHFR-deficient patients [119,120]. Froese et al. suggested the use of betaine as a treatment option during pregnancy since betaine supplementation increased offspring survival and decreased male infertility in a well-characterised gene knock-out mouse model used to study *MTHFR* deficiency [3].

Interestingly, folate stabilises enzyme activity in individuals with the C677T polymorphism, effectively neutralising effects of the polymorphism [7,121]. Jacques et al. reported that the association between the C677T polymorphism and hyperhomocysteinemia was present in patients with low folate levels [121]. This research suggests that folate and vitamin B12 supplementation may effectively treat hyperhomocysteinemia. Guenther et al. heated lymphocyte extracts with or without 5-methyl THF and observed that MTHFR activity was greater in samples heated with folate supplementation, indicating a protective effect on MTHFR by folate [4]. Shi et al. suggested the use of vitamin therapy, possibly in conjunction with medical foods, to improve one-carbon cycle functioning and prevent the onset and progression of DR [43].

## 7. Conclusions and Recommendations

**Figure 2:**
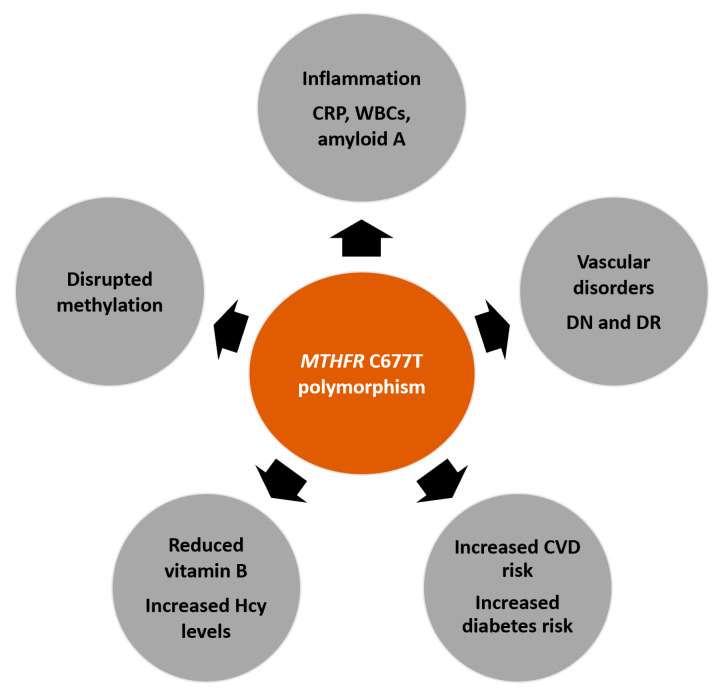
Potential effects of the *MTHFR* C677T polymorphism on several disease states. The polymorphism may affect inflammation, which is identified by increased inflammatory markers, such as c-reactive protein (CRP), white blood cell (WBC) count, and amyloid-A. Many studies have reported associations between the *MTHFR* C677T polymorphism and the development or progression of diabetes, cardiovascular disease (CVD), and vascular disorders (such as diabetic nephropathy (DN) and diabetic retinopathy (DR)). Furthermore, reduced enzyme activity contributes to increased homocysteine (Hcy) levels, reduced B vitamin levels, and disrupted methylation reactions.

Polymorphisms in the *MTHFR* gene may play a role in the development of CVDs and diabetes-associated disorders, such as retinopathy and nephropathy (Figure 2). Research shows that this influence is dependent on ethnicity and geographic location. The evidence should be carefully considered when concluding the association of *MTHFR* with CVD and diabetes development. However, too many studies have reported on positive associations to ignore the potential use of this genetic polymorphism as a screening tool to determine the development and progression of several disease states. Further studies should be conducted with larger cohorts in various geographic regions and with various ethnic groups to successfully conclude the relationship between this gene and cardiovascular risk.

## Figures and Tables

**Table 1:** Further Reading: Meta-analysis studies conducted regarding *MTHFR* polymorphisms in various ethnic groups and associated metabolic diseases.

Study Title	Associated Disease	Reference
*MTHFR* 677T variant contributes to diabetic nephropathy risk in Caucasian individuals with type 2 diabetes: a meta-analysis	T2DM	[59]
Association of *MTHFR* C677T polymorphism and type 2 diabetes mellitus (T2DM) susceptibility	T2DM	[74]
The effect of *MTHFR* C677T polymorphism on type 2 diabetes mellitus with vascular complications in Chinese Han population: a meta-analysis	T2DM and vascular complications	[75]
Methylenetrahydrofolate Reductase Gene C677T Polymorphism and Diabetic Retinopathy: a Meta-Analysis	DR	[76]
Methylenetetrahydrofolate reductase A1298C polymorphism and diabetes risk: evidence from a meta-analysis	T2DM	[77]
Association between *MTHFR* C677T polymorphism and diabetic nephropathy in the Chinese population: An updated meta-analysis and review	DN	[78]
Methylenetetrahydrofolate reductase (*MTHFR*) C677T polymorphism and susceptibility to diabetic nephropathy in Chinese type 2 diabetic patients: a meta-analysis	T2DM and DN	[79]
Increasing prevalence of gestational diabetes mellitus when carrying the T variant allele of the *MTHFR* gene C677T polymorphism: a systematic review and meta-analysis	Gestational diabetes	[80]
Relationship between methylenetetrahydrofolate reductase (*MTHFR*) A1298C gene polymorphism and type 2 diabetic nephropathy risk: a meta-analysis	T2DM and DN	[81]
A Meta-Analysis of Association between Methylenetetrahydrofolate Reductase Gene (*MTHFR*) 677C/T Polymorphism and Diabetic Retinopathy	DR	[82]
Genetics of diabetic neuropathy: Systematic review, meta-analysis and trial sequential analysis	DN	[83]
Methylenetetrahydrofolate reductase C677T polymorphism and type 2 diabetes mellitus in Chinese population: a meta-analysis of 29 case-control studies	T2DM	[84]
Plausible relationship between homocysteine and obesity risk via *MTHFR* gene: a meta-analysis of 38,317 individuals implementing Mendelian randomization	Obesity risk	[85]
Association of homocysteine with type 2 diabetes: a meta-analysis implementing Mendelian randomization approach	T2DM	[86]
Effects of Common Polymorphisms in the *MTHFR* and ACE Genes on Diabetic Peripheral Neuropathy Progression: a Meta-Analysis	DN	[87]
ACE I/D and *MTHFR* C677T polymorphisms are significantly associated with type 2 diabetes in Arab ethnicity: a meta-analysis	T2DM	[88]
Methylenetetrahydrofolate reductase (*MTHFR*) C677T gene polymorphism and diabetic nephropathy susceptibility in patients with type 2 diabetes mellitus	T2DM and DN	[89]
Association between *MTHFR* C677T polymorphism and diabetic nephropathy or diabetes mellitus risk: need for clarification of data in a recent meta-analysis	T2DM and DN	[90]
Methylenetetrahydrofolate reductase C677T polymorphism and diabetic retinopathy risk: a meta-analysis of the Chinese population	DR	[91]
The relationship between methylenetetrahydrofolate reductase C677T polymorphism and diabetic retinopathy: A meta-analysis in multi-ethnic groups	DR	[92]
*MTHFR* gene C677T polymorphism and type 2 diabetic nephropathy in Asian populations: a meta-analysis	DN	[93]
Genetic susceptibility to type 2 diabetes: a global meta-analysis studying the genetic differences in Tunisian populations	T2DM	[94]
Genetic risk of type 2 diabetes in populations of the African continent: A systematic review and meta-analyses	T2DM	[95]
An updated meta-analysis of methylenetetrahydrofolate reductase gene 677C/T polymorphism with diabetic nephropathy and diabetic retinopathy	DR and DN	[96]
Methylenetetrahydrofolate reductase genetic polymorphism and the risk of diabetic nephropathy in type 2 diabetic patients	T2DM and DN	[97]
Common variants of homocysteine metabolism pathway genes and risk of type 2 diabetes and related traits in Indians	T2DM	[98]
Is C677T polymorphism in methylenetetrahydrofolate reductase gene a risk factor for diabetic nephropathy or diabetes mellitus in a Chinese population?	T2DM and DN	[99]
Interactions among Candidate Genes Selected by Meta-Analyses Resulting in Higher Risk of Ischemic Stroke in a Chinese Population	Ischemic stroke	[100]
No Evidence of a Causal Relationship between Plasma Homocysteine and Type 2 Diabetes: A Mendelian Randomization Study	T2DM	[101]

T2DM, type 2 diabetes mellitus; DN, diabetic nephropathy; DR, diabetic retinopathy.

## Data Availability

Not applicable.

## References

[1] Goyette P., Sumner J.S., Milos R., Duncan A.M.V., Rosenblatt D.S., Matthews R.G., Rozen R. (1994). Human methylenetetrahydrofolate reductase: Isolation of cDNA, mapping and mutation identification. Nat. Genet..

[2] Leclerc D., Sibani S., Rozen R. (2015). Molecular Biology of Methylenetetrahydrofolate Reductase (MTHFR) and Overview of Mutations/Polymorphisms. Madame Curie Bioscience Database.

[3] Froese D.S., Huemer M., Suormala T., Burda P., Coelho D., Guéant J.L., Landolt M.A., Kožich V., Fowler B., Baumgartner M.R. (2016). Mutation Update and Review of Severe Methylenetetrahydrofolate Reductase Deficiency. Hum. Mutat..

[4] Guenther B.D., Sheppard C.A., Tran P., Rozen R., Matthews R.G., Ludwig M.L. (1999). The structure and properties of methylenetetrahydrofolate reductase from Escherichia coli suggest how folate ameliorates human hyperhomocysteinemia. Nat. Struct. Biol..

[5] Mudd S.H., Uhlendorf B.W., Freeman J.M., Finkelstein J.D., Shih V.E. (1972). Homocystinuria associated with decreased methylenetetrahydrofolate reductase activity. Biochem. Biophys. Res. Commun..

[6] Kang S.S., Zhou J., Wong P.W., Kowalisyn J., Strokosch G. (1988). Intermediate homocysteinemia: A thermolabile variant of methylenetetrahydrofolate reductase. Am. J. Hum. Genet..

[7] Frosst P., Blom H.J., Milos R., Goyette P., Sheppard C.A., Matthews R.G., Boers G.J., den Heijer M., Kluijtmans L.A., van den Heuvel L.P. (1995). A candidate genetic risk factor for vascular disease: A common mutation in methylenetetrahydrofolate reductase. Nat. Genet..

[8] Goyette P., Pai A., Milos R., Frosst P., Tran P., Chen Z., Chan M., Rozen R. (1998). Gene structure of human and mouse methylenetetrahydrofolate reductase (MTHFR). Mamm. Genome.

[9] Rosenberg N., Murata M., Ikeda Y., Opare-Sem O., Zivelin A., Geffen E., Seligsohn U. (2002). The frequent 5,10-methylenetetrahydrofolate reductase C677T polymorphism is associated with a common haplotype in whites, Japanese, and Africans. Am. J. Hum. Genet..

[10] Rozen R. (1997). Genetic predisposition to hyperhomocysteinemia: Deficiency of methylenetetrahydrofolate reductase (MTHFR). Thromb. Haemost..

[11] Kang S.S., Wong P.W., Susmano A., Sora J., Norusis M., Ruggie N. (1991). Thermolabile methylenetetrahydrofolate reductase: An inherited risk factor for coronary artery disease. Am. J. Hum. Genet..

[12] Botto L.D., Yang Q. (2000). 5,10-Methylenetetrahydrofolate reductase gene variants and congenital anomalies: A HuGE review. Am. J. Epidemiol..

[13] Wilcken B., Bamforth F., Li Z., Zhu H., Ritvanen A., Renlund M., Stoll C., Alembik Y., Dott B., Czeizel A.E. (2003). Geographical and ethnic variation of the 677C > T allele of 5,10 methylenetetrahydrofolate reductase (MTHFR): Findings from over 7000 newborns from 16 areas world wide. J. Med. Genet..

[14] Schneider J.A., Rees D.C., Liu Y.T., Clegg J.B. (1998). Worldwide distribution of a common methylenetetrahydrofolate reductase mutation. Am. J. Hum. Genet..

[15] Bahadır A., Eroz R., Türker Y. (2015). Does the MTHFR C677T gene polymorphism indicate cardiovascular disease risk in type 2 diabetes mellitus patients?. Anatol. J. Cardiol..

[16] Xia L.Z., Liu Y., Xu X.Z., Jiang P.C., Ma G., Bu X.F., Zhang Y., Yu F., Xu K.-S., Li H. (2014). Methylenetetrahydrofolate reductase C677T and A1298C polymorphisms and gastric cancer susceptibility. World J. Gastroenterol..

[17] Lawrence T. (2009). The nuclear factor NF-kappaB pathway in inflammation. Cold Spring Harb. Perspect. Biol..

[18] Hayden M.S., Ghosh S. (2012). NF-κB, the first quarter-century: Remarkable progress and outstanding questions. Genes Dev..

[19] Chen L., Deng H., Cui H., Fang J., Zuo Z., Deng J., Li Y., Wang X., Zhao L. (2017). Inflammatory responses and inflammation-associated diseases in organs. Oncotarget.

[20] Sproston N.R., Ashworth J.J. (2018). Role of C-Reactive Protein at Sites of Inflammation and Infection. Front. Immunol..

[21] Hage F.G., Szalai A.J. (2007). C-reactive protein gene polymorphisms, C-reactive protein blood levels, and cardiovascular disease risk. J. Am. Coll. Cardiol..

[22] Araki A., Hosoi T., Orimo H., Ito H. (2005). Association of plasma homocysteine with serum interleukin-6 and C-peptide levels in patients with type 2 diabetes. Metabolism.

[23] Dedoussis G.V.Z., Panagiotakos D.B., Pitsavos C., Chrysohoou C., Skoumas J., Choumerianou D., Stefanadis C. (2005). An association between the methylenetetrahydrofolate reductase (MTHFR) C677T mutation and inflammation markers related to cardiovascular disease. Int. J. Cardiol..

[24] Leclerc D., Christensen K.E., Cauvi O., Yang E., Fournelle F., Bahous R.H., Malysheva O.V., Deng L., Wu Q., Zhou Z. (2019). Mild Methylenetetrahydrofolate Reductase Deficiency Alters Inflammatory and Lipid Pathways in Liver. Mol. Nutr. Food Res..

[25] Loomba R., Sanyal A.J. (2013). The global NAFLD epidemic. Nat. Rev. Gastroenterol. Hepatol..

[26] An X., Yang Z., An Z. (2017). MiR-149 Compromises the Reactions of Liver Cells to Fatty Acid via its Polymorphism and Increases Non-Alcoholic Fatty Liver Disease (NAFLD) Risk by Targeting Methylene Tetrahydrofolate Reductase (MTHFR). Med. Sci. Monit. Int. Med. J. Exp. Clin. Res..

[27] Bala S., Petrasek J., Mundkur S., Catalano D., Levin I., Ward J., Alao H., Kodys K., Szabo G. (2012). Circulating microRNAs in exosomes indicate hepatocyte injury and inflammation in alcoholic, drug-induced, and inflammatory liver diseases. Hepatology.

[28] Del Campo J.A., Gallego-Durán R., Gallego P., Grande L. (2018). Genetic and Epigenetic Regulation in Nonalcoholic Fatty Liver Disease (NAFLD). Int. J. Mol. Sci..

[29] Liu C.H., Ampuero J., Gil-Gómez A., Montero-Vallejo R., Rojas Á., Muñoz-Hernández R., Gallego-Durán R., Romero-Gómez M. (2018). miRNAs in patients with non-alcoholic fatty liver disease: A systematic review and meta-analysis. J. Hepatol..

[30] Kim T.H., Lee Y., Lee Y.S., Gim J.A., Ko E., Yim S.Y., Jung Y.K., Kang S., Kim M.Y., Kim H. (2021). Circulating miRNA is a useful diagnostic biomarker for nonalcoholic steatohepatitis in nonalcoholic fatty liver disease. Sci. Rep..

[31] Vasku V., Bienertova-Vasku J., Necas M., Vasku A. (2009). MTHFR (methylenetetrahydrofolate reductase) C677T polymorphism and psoriasis. Clin. Exp. Med..

[32] Mahmud N., Molloy A., McPartlin J., Corbally R., Whitehead A.S., Scott J.M., Weir D.G. (1999). Increased prevalence of methylenetetrahydrofolate reductase C677T variant in patients with inflammatory bowel disease, and its clinical implications. Gut.

[33] Yuan Y., Shao W., Li Y. (2017). Associations between C677T and A1298C polymorphisms of MTHFR and susceptibility to rheumatoid arthritis: A systematic review and meta-analysis. Rheumatol. Int..

[34] Cen H., Huang H., Zhang L.N., Liu L.Y., Zhou L., Xin X.F., Zhuo R.J. (2017). Associations of methylenetetrahydrofolate reductase (MTHFR) C677T and A1298C polymorphisms with genetic susceptibility to rheumatoid arthritis: A meta-analysis. Clin. Rheumatol..

[35] Donath M.Y., Böni-Schnetzler M., Ellingsgaard H., Ehses J.A. (2009). Islet inflammation impairs the pancreatic beta-cell in type 2 diabetes. Physiology.

[36] Wellen K.E., Hotamisligil G.S. (2005). Inflammation, stress, and diabetes. J. Clin. Investig..

[37] Khalighi K., Cheng G., Mirabbasi S., Khalighi B., Wu Y., Fan W. (2018). Opposite impact of Methylene tetrahydrofolate reductase C677T and Methylene tetrahydrofolate reductase A1298C gene polymorphisms on systemic inflammation. J. Clin. Lab. Anal..

[38] Clare C.E., Brassington A.H., Kwong W.Y., Sinclair K.D. (2019). One-Carbon Metabolism: Linking Nutritional Biochemistry to Epigenetic Programming of Long-Term Development. Annu. Rev. Anim. Biosci..

[39] Lyon P., Strippoli V., Fang B., Cimmino L. (2020). B Vitamins and One-Carbon Metabolism: Implications in Human Health and Disease. Nutrients.

[40] Newman A.C., Maddocks O.D.K. (2017). One-carbon metabolism in cancer. Br. J. Cancer.

[41] Froese D.S., Fowler B., Baumgartner M.R. (2019). Vitamin B(12), folate, and the methionine remethylation cycle-biochemistry, pathways, and regulation. J. Inherit. Metab. Dis..

[42] Hiraoka M., Kagawa Y. (2017). Genetic polymorphisms and folate status. Congenit. Anom..

[43] Shi C., Wang P., Airen S., Brown C., Liu Z., Townsend J.H., Wang J., Jiang H. (2020). Nutritional and medical food therapies for diabetic retinopathy. Eye Vis..

[44] Alizadeh S., Djafarian K., Moradi S., Shab-Bidar S. (2016). C667T and A1298C polymorphisms of methylenetetrahydrofolate reductase gene and susceptibility to myocardial infarction: A systematic review and meta-analysis. Int. J. Cardiol..

[45] Xuan C., Bai X.Y., Gao G., Yang Q., He G.W. (2011). Association between polymorphism of methylenetetrahydrofolate reductase (MTHFR) C677T and risk of myocardial infarction: A meta-analysis for 8,140 cases and 10,522 controls. Arch. Med. Res..

[46] De Martinis M., Sirufo M.M., Nocelli C., Fontanella L., Ginaldi L. (2020). Hyperhomocysteinemia is Associated with Inflammation, Bone Resorption, Vitamin B12 and Folate Deficiency and MTHFR C677T Polymorphism in Postmenopausal Women with Decreased Bone Mineral Density. Int. J. Environ. Res. Public Health.

[47] Almeida M., Soares M., Fonseca-Moutinho J., Ramalhinho A.C., Breitenfeld L. (2021). Influence of Estrogenic Metabolic Pathway Genes Polymorphisms on Postmenopausal Breast Cancer Risk. Pharmaceuticals.

[48] Sung N.J., Park S.A. (2019). Effect of Natural Compounds on Catechol Estrogen-Induced Carcinogenesis. Biomed. Sci. Lett..

[49] Xu J., Boström A.E., Saeed M., Dubey R.K., Waeber G., Vollenweider P., Marques-Vidal P., Mwinyi J., Schiöth H.B. (2017). A genetic variant in the catechol-O-methyl transferase (COMT) gene is related to age-dependent differences in the therapeutic effect of calcium-channel blockers. Medicine.

[50] Hiraku Y., Yamashita N., Nishiguchi M., Kawanishi S. (2001). Catechol estrogens induce oxidative DNA damage and estradiol enhances cell proliferation. Int. J. Cancer.

[51] Kang S.S., Wong P.W., Norusis M. (1987). Homocysteinemia due to folate deficiency. Metabolism.

[52] Frederiksen J., Juul K., Grande P., Jensen G.B., Schroeder T.V., Tybjaerg-Hansen A., Nordestgaard B.G. (2004). Methylenetetrahydrofolate reductase polymorphism (C677T), hyperhomocysteinemia, and risk of ischemic cardiovascular disease and venous thromboembolism: Prospective and case-control studies from the Copenhagen City Heart Study. Blood.

[53] Lima L.M., Carvalho M.D.G., Fernandes A.P., Sabino A.D.P., Loures-Vale A.A., da Fonseca Neto C.P., Garcia J.C.F., Saad J.A., Sousa M.O. (2007). Homocysteine and methylenetetrahydrofolate reductase in subjects undergoing coronary angiography. Arq. Bras. Cardiol..

[54] Wald D.S., Law M., Morris J.K. (2002). Homocysteine and cardiovascular disease: Evidence on causality from a meta-analysis. BMJ.

[55] Pereira A.C., Schettert I.T., Morandini Filho A.A.F., Guerra-Shinohara E.M., Krieger J.E. (2004). Methylenetetrahydrofolate reductase (MTHFR) c677t gene variant modulates the homocysteine folate correlation in a mild folate-deficient population. Clin. Chim. Acta..

[56] McKinnon P.J., Caldecott K.W. (2007). DNA strand break repair and human genetic disease. Annu. Rev. Genom. Hum. Genet..

[57] Kang S., Zhao X., Liu L., Wu W., Zhang D. (2013). Association of the C677T polymorphism in the MTHFR gene with hemorrhagic stroke: A meta-analysis. Genet. Test. Mol. Biomark..

[58] Klerk M., Verhoef P., Clarke R., Blom H.J., Kok F.J., Schouten E.G. (2002). MTHFR 677C-->T polymorphism and risk of coronary heart disease: A meta-analysis. JAMA.

[59] Yang S., Zhang J., Feng C., Huang G. (2013). MTHFR 677T variant contributes to diabetic nephropathy risk in Caucasian individuals with type 2 diabetes: A meta-analysis. Metabolism.

[60] Kluijtmans L.A., van den Heuvel L.P., Boers G.H., Frosst P., Stevens E.M., van Oost B.A., den Heijer M., Trijbels F.J., Rozen R., Blom H.J. (1996). Molecular genetic analysis in mild hyperhomocysteinemia: A common mutation in the methylenetetrahydrofolate reductase gene is a genetic risk factor for cardiovascular disease. Am. J. Hum. Genet..

[61] Zhong J.H., Rodríguez A.C., Yang N.N., Li L.Q. (2013). Methylenetetrahydrofolate reductase gene polymorphism and risk of type 2 diabetes mellitus. PLoS ONE.

[62] Zalavras C.G., Giotopoulou S., Dokou E., Mitsis M., Ioannou H.V., Tzolou A., Kolaitis N., Vartholomatos G. (2002). Lack of association between the C677T mutation in the 5,10-methylenetetrahydrofolate reductase gene and venous thromboembolism in Northwestern Greece. Int. Angiol..

[63] Lu Y., Zhao Y., Liu G., Wang X., Liu Z., Chen B., Hui R. (2002). Factor V gene G1691A mutation, prothrombin gene G20210A mutation, and MTHFR gene C677T mutation are not risk factors for pulmonary thromboembolism in Chinese population. Thromb. Res..

[64] Quéré I., Perneger T.V., Zittoun J., Bellet H., Gris J.C., Daurès J.P., Schved J.F., Mercier E., Laroche J.P., Dauzat M. (2002). Red blood cell methylfolate and plasma homocysteine as risk factors for venous thromboembolism: A matched case-control study. Lancet.

[65] Keijzer M.B.A.J., den Heijer M., Blom H.J., Bos G.M.J., Willems H.P.J., Gerrits W.B.J., Rosendaal F.R. (2002). Interaction between hyperhomocysteinemia, mutated methylenetetrahydrofolatereductase (MTHFR) and inherited thrombophilic factors in recurrent venous thrombosis. Thromb. Haemost..

[66] Li X.M., Wei Y.F., Hao H.L., Hao Y.B., He L.S., Li J.D., Mei B., Wang S.Y., Wang C., Wang J.X. (2002). Hyperhomocysteinemia and the MTHFR C677T mutation in Budd-Chiari syndrome. Am. J. Hematol..

[67] Casas J.P., Hingorani A.D., Bautista L.E., Sharma P. (2004). Meta-analysis of genetic studies in ischemic stroke: Thirty-two genes involving approximately 18,000 cases and 58,000 controls. Arch. Neurol..

[68] Tavakkoly Bazzaz J., Shojapoor M., Nazem H., Amiri P., Fakhrzadeh H., Heshmat R., Parvizi M., Hasani Ranjbar S., Amoli M.M. (2010). Methylenetetrahydrofolate reductase gene polymorphism in diabetes and obesity. Mol. Biol. Rep..

[69] Mehri S., Koubaa N., Nakbi A., Hammami S., Chaaba R., Mahjoub S., Zouari B., Abid M., Ben Arab S., Baudin B. (2010). Relationship between genetic polymorphisms of angiotensin-converting enzyme and methylenetetrahydrofolate reductase as risk factors for type 2 diabetes in Tunisian patients. Clin. Biochem..

[70] Pollex R.L., Mamakeesick M., Zinman B., Harris S.B., Hanley A.J.G., Hegele R.A. (2005). Methylenetetrahydrofolate reductase polymorphism 677C > T is associated with peripheral arterial disease in type 2 diabetes. Cardiovasc. Diabetol..

[71] Bentzon J.F., Otsuka F., Virmani R., Falk E. (2014). Mechanisms of plaque formation and rupture. Circ. Res..

[72] Nishio H., Lee M.J., Fujii M., Kario K., Kayaba K., Shimada K., Matsuo M., Sumino K. (1996). A common mutation in methylenetetrahydrofolate reductase gene among the Japanese population. Jpn. J. Hum. Genet..

[73] Qian X., Lu Z., Tan M., Liu H., Lu D. (2007). A meta-analysis of association between C677T polymorphism in the methylenetetrahydrofolate reductase gene and hypertension. Eur. J. Hum. Genet..

[74] Meng Y., Liu X., Ma K., Zhang L., Lu M., Zhao M., Guan M.-X., Qin G. (2019). Association of MTHFR C677T polymorphism and type 2 diabetes mellitus (T2DM) susceptibility. Mol. Genet. Genom. Med..

[75] Zhang D., Zhou Y., Han L., Ji H., Li J. (2014). The effect of MTHFR C677T polymorphism on type 2 diabetes mellitus with vascular complications in Chinese Han population: A meta-analysis. Endocr. J..

[76] Shen C., Zhao M., Li Y.Y., Liu N.P. (2020). Methylenetrahydrofolate Reductase Gene C677T Polymorphism and Diabetic Retinopathy: A Meta-Analysis. Chin. Med. Sci. J..

[77] Yan Y., Liang H., Yang S., Wang J., Xie L., Qin X., Li S. (2014). Methylenetetrahydrofolate reductase A1298C polymorphism and diabetes risk: Evidence from a meta-analysis. Ren. Fail..

[78] Xiong X., Lin X.K., Xiao X., Qin D.P., Zhou D.Y., Hu J.G., Liu Y., Zhong X.S. (2016). Association between MTHFR C677T polymorphism and diabetic nephropathy in the Chinese population: An updated meta-analysis and review. Nephrology.

[79] Chang W., Zhang L., Yao Y., Su H., Jin Y., Chen Y. (2013). Methylenetetrahydrofolate reductase (MTHFR) C677T polymorphism and susceptibility to diabetic nephropathy in Chinese type 2 diabetic patients: A meta-analysis. Ren. Fail..

[80] Chen Y., Lu M., Nie J., Liu J., Liu Y., Meng Y., Sun X., Ji C., Zhang J., Yang X. (2021). Increasing prevalence of gestational diabetes mellitus when carrying the T variant allele of the MTHFR gene C677T polymorphism: A systematic review and meta-analysis. Arch. Gynecol. Obstet..

[81] Zhang J., Xiao Y., Zhang X.W., Gao Z.Q., Han J.H. (2014). Relationship between methylenetetrahydrofolate reductase (MTHFR) A1298C gene polymorphism and type 2 diabetic nephropathy risk: A meta-analysis. Ren. Fail..

[82] Luo S., Wang F., Shi C., Wu Z. (2016). A Meta-Analysis of Association between Methylenetetrahydrofolate Reductase Gene (MTHFR) 677C/T Polymorphism and Diabetic Retinopathy. Int. J. Environ. Res. Public Health.

[83] Zhao Y., Zhu R., Wang D., Liu X. (2019). Genetics of diabetic neuropathy: Systematic review, meta-analysis and trial sequential analysis. Ann. Clin. Transl. Neurol..

[84] Zhu B., Wu X., Zhi X., Liu L., Zheng Q., Sun G. (2014). Methylenetetrahydrofolate reductase C677T polymorphism and type 2 diabetes mellitus in Chinese population: A meta-analysis of 29 case-control studies. PLoS ONE.

[85] Fu L., Li Y.N., Luo D., Deng S., Hu Y.Q. (2019). Plausible relationship between homocysteine and obesity risk via MTHFR gene: A meta-analysis of 38,317 individuals implementing Mendelian randomization. Diabetes Metab. Syndr. Obes..

[86] Huang T., Ren J., Huang J., Li D. (2013). Association of homocysteine with type 2 diabetes: A meta-analysis implementing Mendelian randomization approach. BMC Genom..

[87] Wu S., Han Y., Hu Q., Zhang X., Cui G., Li Z., Guan Y. (2017). Effects of Common Polymorphisms in the MTHFR and ACE Genes on Diabetic Peripheral Neuropathy Progression: A Meta-Analysis. Mol. Neurobiol..

[88] Al-Rubeaan K., Siddiqui K., Saeb A.T.M., Nazir N., Al-Naqeb D., Al-Qasim S. (2013). ACE I/D and MTHFR C677T polymorphisms are significantly associated with type 2 diabetes in Arab ethnicity: A meta-analysis. Gene.

[89] Zhou T.B., Drummen G.P.C., Jiang Z.P., Li H.Y. (2015). Methylenetetrahydrofolate reductase (MTHFR) C677T gene polymorphism and diabetic nephropathy susceptibility in patients with type 2 diabetes mellitus. Ren. Fail..

[90] Chang W.W., Jin Y.L., Zhang L., Chen Y., Yao Y.S. (2012). Association between MTHFR C677T polymorphism and diabetic nephropathy or diabetes mellitus risk: Need for clarification of data in a recent meta-analysis. Arch. Med. Res..

[91] Xu W.H., Zhuang Y., Han X., Yuan Z.L. (2020). Methylenetetrahydrofolate reductase C677T polymorphism and diabetic retinopathy risk: A meta-analysis of the Chinese population. J. Int. Med. Res..

[92] Chen D., Wang J., Dan Z., Shen X., Ci D. (2018). The relationship between methylenetetrahydrofolate reductase C677T polymorphism and diabetic retinopathy: A meta-analysis in multiethnic groups. Ophthalmic Genet..

[93] Chen H., Wei F., Wang L., Wang Z., Meng J., Jia L., Sun G., Zhang R., Li B., Yu H. (2015). MTHFR gene C677T polymorphism and type 2 diabetic nephropathy in Asian populations: A meta-analysis. Int. J. Clin. Exp. Med..

[94] Berhouma R., Kouidhi S., Ammar M., Abid H., Baroudi T., Ennafaa H., Benammar-Elgaaied A. (2012). Genetic susceptibility to type 2 diabetes: A global meta-analysis studying the genetic differences in Tunisian populations. Hum. Biol..

[95] Yako Y.Y., Guewo-Fokeng M., Balti E.V., Bouatia-Naji N., Matsha T.E., Sobngwi E., Erasmus R.T., Echouffo-Tcheugui J.B., Kengne A.P. (2016). Genetic risk of type 2 diabetes in populations of the African continent: A systematic review and meta-analyses. Diabetes Res. Clin. Pract..

[96] Niu W., Qi Y. (2012). An updated meta-analysis of methylenetetrahydrofolate reductase gene 677C/T polymorphism with diabetic nephropathy and diabetic retinopathy. Diabetes Res. Clin. Pract..

[97] Guan H., Xia M.D., Wang M., Guan Y.J., Lyu X.C. (2020). Methylenetetrahydrofolate reductase genetic polymorphism and the risk of diabetic nephropathy in type 2 diabetic patients. Medicine.

[98] Chauhan G., Kaur I., Tabassum R., Dwivedi O.P., Ghosh S., Tandon N., Bharadwaj D. (2012). Common variants of homocysteine metabolism pathway genes and risk of type 2 diabetes and related traits in Indians. Exp. Diabetes Res..

[99] Cui W., Du B., Jia Y., Zhou W., Liu S., Cui Y., Ma F., Luo P., Miao L. (2012). Is C677T polymorphism in methylenetetrahydrofolate reductase gene a risk factor for diabetic nephropathy or diabetes mellitus in a Chinese population?. Arch. Med. Res..

[100] Luo M., Li J., Sun X., Lai R., Wang Y., Xu X., Sheng W. (2015). Interactions among Candidate Genes Selected by Meta-Analyses Resulting in Higher Risk of Ischemic Stroke in a Chinese Population. PLoS ONE.

[101] Kumar J., Ingelsson E., Lind L., Fall T. (2015). No Evidence of a Causal Relationship between Plasma Homocysteine and Type 2 Diabetes: A Mendelian Randomization Study. Front. Cardiovasc. Med..

[102] Bird A. (2002). DNA methylation patterns and epigenetic memory. Genes Dev..

[103] Portela A., Esteller M. (2010). Epigenetic modifications and human disease. Nat. Biotechnol..

[104] Narayanan N., Tyagi N., Shah A., Pagni S., Tyagi S.C. (2013). Hyperhomocysteinemia during aortic aneurysm, a plausible role of epigenetics. Int. J. Physiol. Pathophysiol. Pharmacol..

[105] Dayeh T., Volkov P., Salö S., Hall E., Nilsson E., Olsson A.H., Kirkpatrick C.L., Wollheim C.B., Eliasson L., Rönn T. (2014). Genome-wide DNA methylation analysis of human pancreatic islets from type 2 diabetic and non-diabetic donors identifies candidate genes that influence insulin secretion. PLoS Genet..

[106] Pheiffer C., Erasmus R.T., Kengne A.P., Matsha T.E. (2016). Differential DNA methylation of microRNAs within promoters, intergenic and intragenic regions of type 2 diabetic, pre-diabetic and non-diabetic individuals. Clin. Biochem..

[107] Volkmar M., Dedeurwaerder S., Cunha D.A., Ndlovu M.N., Defrance M., Deplus R., Calonne E., Volkmar U., Igoillo-Esteve M., Naamane N. (2012). DNA methylation profiling identifies epigenetic dysregulation in pancreatic islets from type 2 diabetic patients. EMBO J..

[108] Matsha T.E., Pheiffer C., Mutize T., Erasmus R.T., Kengne A.P. (2016). Glucose Tolerance, MTHFR C677T and NOS3 G894T Polymorphisms, and Global DNA Methylation in Mixed Ancestry African Individuals. J. Diabetes Res..

[109] Abbas S., Raza S.T., Ahmed F., Ahmad A., Rizvi S., Mahdi F. (2013). Association of genetic polymorphism of PPARγ-2, ACE, MTHFR, FABP-2 and FTO genes in risk prediction of type 2 diabetes mellitus. J. Biomed. Sci..

[110] El Hajj Chehadeh S.W., Jelinek H.F., Al Mahmeed W.A., Tay G.K., Odama U.O., Elghazali G.E.B., Al Safar H.S. (2016). Relationship between MTHFR C677T and A1298C gene polymorphisms and complications of type 2 diabetes mellitus in an Emirati population. Meta Gene.

[111] Calderon G.D., Juarez O.H., Hernandez G.E., Punzo S.M., De la Cruz Z.D. (2017). Oxidative stress and diabetic retinopathy: Development and treatment. Eye.

[112] Yokoyama H., Okudaira M., Otani T., Sato A., Miura J., Takaike H., Yamada H., Muto K., Uchigata Y., Ohashi Y. (2000). Higher incidence of diabetic nephropathy in type 2 than in type 1 diabetes in early-onset diabetes in Japan. Kidney Int..

[113] Ramanathan G., Harichandana B., Kannan S., Elumalai R., Sfd P. (2019). Association between end-stage diabetic nephropathy and MTHFR (C677T and A1298C) gene polymorphisms. Nephrology.

[114] Teschner M., Kosch M., Schaefer R.M. (2002). Folate metabolism in renal failure. Nephrol. Dial. Transplant..

[115] Errera F.I.V., Silva M.E.R., Yeh E., Maranduba C.M.C., Folco B., Takahashi W., Pereira A.C., Krieger J.E., Passos-Bueno M.R. (2006). Effect of polymorphisms of the MTHFR and APOE genes on susceptibility to diabetes and severity of diabetic retinopathy in Brazilian patients. Braz. J. Med. Biol. Res..

[116] Dos Santos Nunes M.K., Silva A.S., de Queiroga Evangelista I.W., Filho J.M., Gomes C.N.A.P., do Nascimento R.A.F., Luna R.C.P., de Carvalho Costa M.J., de Oliveira N.F.P., Persuhn D.C. (2017). Hypermethylation in the promoter of the MTHFR gene is associated with diabetic complications and biochemical indicators. Diabetol. Metab. Syndr..

[117] Whitehead A.S., Gallagher P., Mills J.L., Kirke P.N., Burke H., Molloy A.M., Weir D.G., Shields D.C., Scott J.M. (1995). A genetic defect in 5,10 methylenetetrahydrofolate reductase in neural tube defects. QJM.

[118] Ou C.Y., Stevenson R.E., Brown V.K., Schwartz C.E., Allen W.P., Khoury M.J., Rozen R., Oakley G.P.J., Adams M.J.J. (1996). 5,10 Methylenetetrahydrofolate reductase genetic polymorphism as a risk factor for neural tube defects. Am. J. Med. Genet..

[119] Diekman E.F., de Koning T.J., Verhoeven-Duif N.M., Rovers M.M., van Hasselt P.M. (2014). Survival and psychomotor development with early betaine treatment in patients with severe methylenetetrahydrofolate reductase deficiency. JAMA Neurol..

[120] Strauss K.A., Morton D.H., Puffenberger E.G., Hendrickson C., Robinson D.L., Wagner C., Stabler S.P., Allen R.H., Chwatko G., Jakubowski H. (2007). Prevention of brain disease from severe 5,10-methylenetetrahydrofolate reductase deficiency. Mol. Genet. Metab..

[121] Jacques P.F., Bostom A.G., Williams R.R., Ellison R.C., Eckfeldt J.H., Rosenberg I.H., Selhub J., Rozen R. (1996). Relation between folate status, a common mutation in methylenetetrahydrofolate reductase, and plasma homocysteine concentrations. Circulation.

